# Assessment and Development of the Antifungal Agent Caspofungin for Aerosolized Pulmonary Delivery

**DOI:** 10.3390/pharmaceutics13040504

**Published:** 2021-04-07

**Authors:** Iching G. Yu, David M. Ryckman

**Affiliations:** Trilogy Therapeutics, Inc., San Diego, CA 92130, USA; gyu@trilogytherapeutics.com

**Keywords:** inhalation, pulmonary drug delivery, caspofungin, antifungal, formulation, peptide(s), chemical stability, solid-state stability, pharmacokinetic/pharmacodynamic (PK/PD) correlation

## Abstract

Invasive Pulmonary Aspergillosis (IPA) and *Pneumocystis jiroveci* Pneumonia (PCP) are serious fungal pulmonary diseases for immunocompromised patients. The brand name drug CANCIDAS^®^ (Caspofungin acetate for injection) is FDA approved to treat IPA, but is only 40% effective. Efficacious drug levels at the lung infection site are not achieved by systemic administration. Increasing the dose leads to toxicity. The objective, here, is to reformulate caspofungin for aerosolization to high drug concentration by lung targeted delivery and avoid systemic distribution. Described in this paper is a new, room temperature-stable formulation that meets these goals. The in vitro antifungal activity, solid state and reconstituted stability, and aerosol properties of the new formulation are presented. In addition, pharmacokinetic parameters and tissue distribution data are determined from nose-only inhalation studies in rats. Plasma and tissue samples were analyzed by High Performance Liquid Chromatography-tandem Mass Spectrometry (HPLC-MS-MS). Inhaled drug concentrations for caspofungin Active Pharmaceutical Ingredient (API), and the new formulation, were compared at the same dose. In the lungs, the parameters C_max_ and Area Under Curve (AUC) showed a 70%, and 60%, respective increase in drug deposition for the new formulation without significant systemic distribution. Moreover, the calculated pharmacodynamic indices suggest an improvement in efficacy. These findings warrant further animal toxicology studies and human clinical trials, with inhaled caspofungin, for treating IPA.

## 1. Introduction

Fungal pulmonary infections, caused by *Aspergillus* or *Pneumocystis jiroveci*, are serious diseases that devastate immunocompromised patients who receive chemotherapy or immunosuppressive agents, associated with their illness, as well as HIV patients. Ubiquitous airborne spores are inhaled and become established in the lung [1]. The spores germinate and lead to hyphal growth that produce the conidiophores, which in turn, generate additional spores that continue to exponentially reproduce the cycle. For healthy populations, alveolar macrophages in the lung remove these spores efficiently. Patients with compromised immune systems, due to chemotherapy or immunosuppressive agents, cannot eradicate spores like healthy individuals. *Aspergillus* propagates very rapidly in these patients’ lungs. One multicenter study found that, after an IPA diagnosis, the medium time to death was 16 days [2]. The overall case fatality rate, even with the treatment, can reach 86% in bone marrow transplant patients [3]. Despite the high mortality of *Aspergillus* infections, current systemic IPA treatments only provide modest efficacy for all patients averaging between 40–50%.

Morphologically *Aspergillus* grows and propagates within the lung cavity. Data from the Prospective Antifungal Therapy Alliance (PATH Alliance) registry indicated that 76% of patients had *Aspergillus* infection confined exclusively in the lung [4]; another study showed 89% of patients had exclusive pulmonary infection [2]. In a previous animal study with the antifungal agent caspofungin showed that only a small percentage (5%) of drug delivered intravenously to rats was detected in the lung tissues [5]. This may explain low human efficacy (40%) of caspofungin in treating IPA. Drug concentration in lung may be more relevant to human efficacy than plasma concentrations. It is likely that an inhaled approach with caspofungin can increase drug concentration in the lung and could make a positive impact on human efficacy. Further advocacy for this approach was published in a recent letter to the *International Journal of Infectious Diseases*, in order to treat *Pneumocystis jiroveci* Pneumonia (PCP) with inhaled caspofungin [6]. An inhaled caspofungin formulation could be used to treat fungal pulmonary infections, such as IPA and PCP.

Caspofungin is the first in class echinocandin and shows superior in vitro activity against *Aspergillus* among the three classes of antifungals namely polyenes, azoles and echinocandins [5]. Caspofungin specifically targets the tips and branch points of the growing hyphae, the site of β-(1,3)-d-glucan synthase that form the major component of the *Aspergillus* and *Pneumocystis jiroveci* cell wall. Here, it disrupts the integrity of the fungal cell wall, which loses its mechanical strength, and becomes destroyed by intracellular osmotic pressure. A topical application of the infection site may provide superior efficacy, as observed in the in vitro studies.

Caspofungin for injection (tradename: CANCIDAS^®^) is sold by Merck & Co., Inc. The approved CANCIDAS^®^ dose is a 70 mg loading dose and then 50 mg/daily. Previous intravenous (IV) studies with caspofungin in animals have shown that IV administered drug only delivers about 5% of the drug level in blood to the lungs. Moreover, the drug level in the lung did not meet pharmacokinetic (PK) and pharmacodynamic (PD) targets required for clinical efficacy [5]. Most of the IV drugs is distributed to other sensitive target organs, such as the liver and kidneys, which if given in higher doses, can lead to toxicities. The low drug distribution to the infection site (lung) may explain why good in vitro activity is not mirrored in clinical efficacy [7]. In a clinical study treating IPA, patients received a caspofungin dose that was three times higher than the approved IV dose. This high dose group (200 mg/day intravenously) showed a 60% response rate (either complete response or partial response), whereas the standard dose group only showed a 44% favorable response [8]. The high dose group showed a 36% improved response rate over the standard dose group, but 65% of patients in the high dose group presented with liver toxicity, including elevated liver enzymes and liver failure. No liver enzyme abnormalities were observed for patients in the standard dose group. It is reasonable to conclude that patients in the high dose group had more drug delivered to the lungs, resulting in more favorable responses, but at the cost of liver toxicity. An ideal approach would be to increase the drug concentration in the lungs without increasing systemic distribution. An inhaled formulation for caspofungin may accomplish this goal.

The CANCIDAS^®^ lyophilized formulation is unstable at room temperature and must be stored at 2–8 °C. It is reconstituted for daily IV use only, to give a 10 mg/mL caspofungin solution. Moreover, this reconstituted solution is only stable for 1 h at ≤ 25°C. The lack of room temperature stability of CANCIDAS^®^ makes it unlikely to be used for inhalation and a room temperature stable formulation is critical for delivering a high dose of caspofungin directly to the lung to achieve better efficacy. Caspofungin stability studies show that the primary degradation route is hydrolysis and subsequent dimerization of the hydrolyzed product, leading to the product failing specification [9]. Protection from water is a concern [9]. Therefore, we have chosen to examine polyvinylpyrrolidone (PVP) as an alternate excipient to the commercial formulation. Povidone or PVP is widely used in the food and cosmetic industries. In addition, PVP is an excipient in many oral and IV drugs and approved for nasal inhalants such as PVP-I_2_ [10]. Active research and development are underway for the use of PVP in metered dose inhalers and spray dried formulation for analgesics and antipyretics [11]. For an inhaled formulation, PVP has several desirable properties, including taste masking for an inhaled formulation. Furthermore, PVP is highly water soluble and can bind to at least 40% of its weight in water while in solid state [12]. Hydrolytic degradation of caspofungin may be inhibited by PVP when it favorably sequestering residual water in the lyophile cake, and protects caspofungin from exposure to water and degradation.

In addition to drug efficacy and mechanism of action, there are several factors to consider when developing an inhaled drug, which include the chemical properties of the drug, stability of the formulation, drug retention time in lung, oral bioavailability and aerosol particle size. Here, we present a new formulation and route of delivery for caspofungin that satisfy these criteria. We also show data that demonstrate the ability to deliver a high dose of drug topically to the lung without widespread systemic distribution.

## 2. Materials and Methods

### 2.1. In Vitro Antifungal Activity with TTI-016, TTI-017 Compared to CANCIDAS^®^ and API

Antifungal potency of the test article(s) was measured using the in vitro broth microdilution assay under test conditions described by the Clinical and Laboratory Standards Institute. Minimum Inhibitory Concentration (MIC) is defined as the lowest concentration of an agent that causes a specified reduction in visible growth of the microorganism. Minimum Effective Concentration (MEC) is defined as the lowest concentration of an agent that leads to the growth of small, rounded, compact hyphal forms, as compared to the hyphal growth typically seen in the growth control well. MIC values were determined for 9 *Candida* spp., and MEC values were determined for filamentous fungus species (*Aspergillus fumigatus* and *Trichophyton rubrum*). Details of the test article preparations and methodologies are described in Supplementary Material S1. CANCIDAS^®^ and caspofungin API were used to compare antifungal potency of laboratory formulation code designations TTI-016 and TTI-017, and Amphotericin B was used as an experimental positive control.

### 2.2. Caspofungin Stability Studies

For stability studies, caspofungin was purchased from BrightGene Bio-Medical Technology Co. Ltd. (Jiangsu, China) and Chunghwa Chemical Synthesis and Biotech Co. Ltd. (New Taipei City, Taiwan). Polyvinylpyrrolidone K30 was purchased from Spectrum Chemicals (Gardena, CA, USA). Lyophilization was accomplished using a VirTis benchtop manifold lyophilizer (SP Scientific, Stone Ridge, NY, USA) under vacuum of <50 mTorr. Details for the lyophilization process and formulation preparations are presented in Supplementary Material S2. High-performance liquid chromatography (HPLC) analysis was carried out on a Waters 2695 separations module (Waters Corporation, Milford, MA, USA) equipped with an autosampler and a Waters 996 photodiode array detector. For impurity determinations a Waters Symmetry (Waters Corporation, Milford, MA, USA) C18 3.5 µm, 4.6 × 75 mm column was used. For weight-weight assay a Waters Symmetry C18 Column (Waters Corporation, Milford, MA, USA), 3.5 µm, 4.6 mm × 100 mm column was employed. Sample preparation, the HPLC conditions, mobile phase and gradients are described in Supplementary Material S2. Reconstituted stability analyses were analyzed using the above HPLC methods. Details of the procedures are described in Supplementary Material S2.

### 2.3. Aerosol Characterization

Aerosol particle size distribution was determined once with a quartz crystal microbalance (QCM) cascade impactor (California Measurements Inc., Sierra Madre, CA, USA) equipped with 10 stages to collect size-segregated samples. The mass median aerodynamic diameter (MMAD) and geometric standard deviation (GSD) were calculated from the mass accumulated on each collection stage of the QCM. Each formulation was tested by aerosolizing it with a PARI LC Star nebulizer (PARI Respiratory Equipment, Inc., Midlothian, VA, USA) connected to compressed air at 28 psi. The air flow to the nebulizer was about 6.2 L/min. Approximately 7 mL of the test formulation solution was placed in the nebulizer, weighed and connected to the air supply for aerosolization. The nebulizer was operated for 12 min, and post weighed. The generated aerosol was delivered into a plenum with a narrow opening. A stream of test aerosol was sampled from the plenum for particle size determination. A Quartz Crystal Microbalance based cascade impactor (TSI Inc., Shoreview, MN, USA) was used for these determinations. Details of the formulation preparations are described in Supplementary Material S3.

### 2.4. Inhalation Pharmacokinetic Studies with Caspofungin Formulation

The study protocols (No. 2635, approval date: 10 May 2016) were approved by the institutional review board at Illinois Institute of Technology Research Institute (IITRI). The study also complied with all applicable sections of the Animal Welfare Act (AWA; Title 9, Code of Federal Regulations), the Public Health Service Policy on Humane Care and Use of Laboratory Animals (National Institutes of Health’s Office of Laboratory Animal Welfare, 2002), and the Guide for the Care and Use of Laboratory Animals (National Research Council, 2011).

Sprague-Dawley derived male rats [Crl:CD^®^(CD)Br] were obtained from Charles River Laboratories, Inc., Wilmington, MA, USA, for use in this study. The animals were approximately 8.5 weeks old at the start of the first exposure to the test article. The animals were randomized into 15 animals per group based on the body weight. Each group was given a single dose of test article. Caspofungin was administered to rats at a target dose of 2 mg/kg by nose only inhalation (by deposition) or intravenously to determine the plasma and tissue concentrations and pharmacokinetics.

The dose targeted for deposition via inhalation was 2 mg/kg and was calculated based on this equation,

Deposited dose = (C × RMV × T × DF)/BW(1)

where C is the average caspofungin concentration in the exposure atmosphere during the exposure period, RMV is the respiratory minute volume, T is the exposure time, DF is the deposition fraction (assumed to be 10% per FDA guidelines) and BW is the average animal body weight on exposure day.

The dose for IV administration was calculated based on the body weight of each animal:

Delivered dose = W × 2 mg/kg(2)

where W is animal weight (kg).

Whole blood samples were collected from three animals per time-point at approximately 0.5, 1, 2, 4, 8, 12, 24 and 48 h and 7 days after dose administration for plasma drug level determination. Rats were anesthetized with 70% CO2/30% air and blood was collected from the retro-orbital plexus and placed into tubes containing anticoagulant (EDTA). Blood samples were placed on ice immediately following collection and processed (i.e., centrifuged) to plasma. The samples were then stored frozen (at approximately −70 °C) until analyzed. Tissue specimens (lung, liver and kidney) were collected from three animals per time point at 0.5, 2, 24 and 48 h and 7 days after dose administration. All tissue specimens were stored frozen at approximately −70 °C until analyzed.

Test atmosphere generation, test atmosphere monitoring, aerosol particle distribution, test article administration, toxicology methods, and the bioanalytical method and analysis are described in the prior study and Supplementary Material S4 [5].

### 2.5. Statistical Analysis

*t*-test was used to analyze the statistical significance of the difference between groups. A difference of *p* < 0.05 was considered significance.

## 3. Results

Lyophilized formulations of PVP and caspofungin were prepared in order to examine the in vitro activity, stability, aerosol properties, as well as the inhaled pharmacokinetics and distribution in targeted organs. We formulated compositions with 1:1, 2:1 and 4:1 ratios of PVP:caspofungin, using water as the lyophile diluent, but we selected the high 4:1 ratio of PVP:caspofungin for in vitro testing to investigate any abnormalities caused by the PVP. Normal saline and phosphate buffered (PBS) saline were used as the lyophile diluent for TTI-016 and TTI-017. Reconstitution of the lyophilized cakes with normal saline was prepared in both TTI-016 and TTI-017. CANCIDAS^®^ was reconstituted per label instructions and caspofungin API was dissolved in normal saline.

### 3.1. In Vitro Antifungal Activity

Following incubation, the test plates were visually examined and wells were scored for growth or complete growth inhibition to define the MIC values. Microscopy examination was used to determine the MEC values. Each test substance was evaluated in duplicate and the results are the duplicate test values. Vehicle-control and an active reference agent were used as blank and positive controls, respectively. The formulations TTI-016 and TTI-017 retained anti-fungal activity against the tested *Candida*, *Cryptococcus*, *Aspergillus* and *Trichophyton* strain as shown in Table 1.

TTI-016 and TTI-017 have similar antifungal activity (*p* = 0.56 and *p* = 0.34, respectively) compared to CANCIDAS^®^ against *Candida*, *Aspergillus* and *Trichophyton* as shown in Table 1.

### 3.2. Formulation Stability

The stability of the lyophilized formulations was studied under two sets of temperature conditions, 2–5 °C and 25 °C. The frozen solutions were lyophilized for 90 h to reduce the water content of the cake to less than 1.0% (specifically, TTI-016: 0.87% and TTI-017: 0.79% water content). At the specified time points the test articles were reconstituted with normal saline to yield a 10 mg/mL solution for HPLC assay. For comparison, we also lyophilized caspofungin API alone. The results of these stability tests are shown in Table 2.

For comparison and shown in Table 3, are the total impurities reported for CANCIDAS formulation versus the total impurities measured for TTI-016 and TTI-017 at the same time points when stored at 25 °C [13]. Both TTI-016 and TTI-017 show significantly less total impurity compared to CANCIDAS formulation (*p* < 0.01). There is not a significant difference in total impurities between TTI-016 and TTI-017 (*p* = 0.73).

As shown in Table 4, and of particular significance, is the reconstituted, room temperature, stability of these lyophilized formulations when compared to reconstituted CANCIDAS^®^. According to the Package Insert, reconstituted 10 mg/mL CANCIDAS^®^ solution is only stable for up to one hour at ≤25 °C. TTI-016 and TTI-017, when reconstituted to the same concentration, remained stable for at least seven hours at room temperature. There is no statistically significant difference in the reconstituted solutions of TTI-016 and TTI-017.

### 3.3. Aerosol Properties

In addition to room temperature stability, an inhaled product must have an appropriate pH, osmolality and aerosol particle size. All the presented formulations (Table 5) met the first two criteria. However, for optimal lung deposition, the aerosol must have a particle size between 1 and 5 microns. Particles smaller than 1 micron can be exhaled and those larger than 5 microns can be swallowed instead of being inhaled. Particle size outside this range results in suboptimal lung deposition [14]. TTI-002 falls just outside of this ideal range with a mass median aerodynamic diameter (MMAD) of 0.89 microns (Table 5). TTI-013 and TTI-016 aerosol particle sizes allow the drug to penetrate deeply into the lung.

### 3.4. Pharmacokinetics

**Figure 1:**
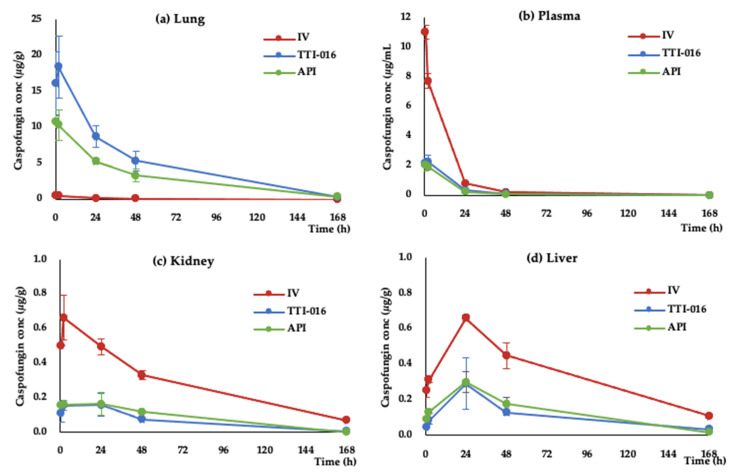
Time-dependent concentration curve in tissues of IV CANCIDAS^®^, inhaled TTI-016 and API cohorts. Caspofungin concentrations were shown in (**a**) lung, (**b**) plasma, (**c**) kidney, and (**d**) liver.

Based on the in vitro activity, solid state and reconstituted stability, and aerosol properties, TTI-016 was selected for further evaluations in the rat inhalation pharmacokinetic studies. Test formulations were prepared, as described and administered at a deposited dose of 2 mg/kg by IV or nose only inhalation. This dose is the nominal effective dose in rodents [15]. Exposure of male rats to IV CANCIDAS^®^ or inhaled caspofungin API and TTI-016 resulted in no test-article-related mortality, no clinical signs of toxicity, no effects on body weight, and no gross necropsy findings attributable to exposure to the test article. Drug distribution in plasma and organs (lung, kidney and liver) were analyzed by HPLC-MS-MS to determine drug concentration and pharmacokinetics. Table 6 present caspofungin concentrations in lung, plasma, kidney and liver of three cohorts. The data are present as mean values (*n* = 3) ± Standard Deviation (SD). The cohort administered TTI-016 showed improved drug deposition in lungs by 70% measured by C_max_ compared to the API cohort without increasing systemic exposure, as shown in Table 7 [5]. AUC was calculated for each animal and presented as mean values (*n* = 3) ± SD. Statistical difference in AUC of each cohort was analyzed using *t*-test. Caspofungin plasma and lung levels in both inhaled API and TTI-016 showed statistical difference from IV CANCIDAS^®^ (*p* < 0.01 and *p* < 0.05, respectively). The time-dependent concentration curves in lungs highlight the difference in drug concentrations in the lung between the two inhaled cohorts (Figure 1a). Drug concentrations in plasma, kidney and liver for the inhaled groups are similar (Figure 1b–d).

**Figure 2:**
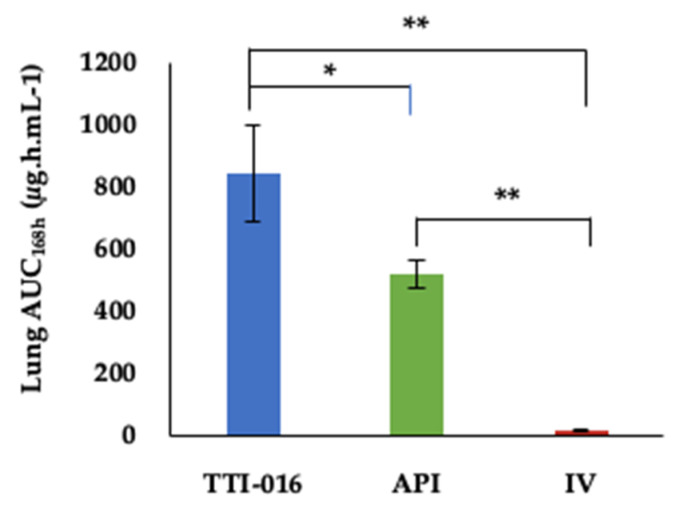
AUC_168 h_ in lung of IV CANCIDAS^®^ and aerosolized TTI-016 and API. The data are presented as the mean values (*n* = 3) ± SD. The asterisk (*) corresponds to significant difference (*p* < 0.05); the double asterisk (**) represents significant difference (*p* < 0.01).

The lung AUC_168h_ data also showed a 62% higher drug exposure in the inhaled TTI-016 cohort compared to the inhaled API cohort (*p* < 0.05) and 4711% higher than the IV group (*p* < 0.01) (Figure 2 and Table 7).

## 4. Discussion

When developing a new formulation for a known drug, it is important to ensure that the drug remains as active as the existing formulation. Testing against the *Candida* spp., *Aspergillus fumigatus* and *Trichophyton rubrum* showed that the new formulations, TTI-016 and TTI-017, retain antifungal activity comparable to that of CANCIDAS^®^ and the presence of formulation excipients does not interfere with antifungal activity (Table 1).

CANCIDAS^®^ must be stored at low temperatures (e.g., 2–8 °C), and must be used within 1 h after reconstitution. The stability studies, presented here, show clear advantages to the new formulations, TTI-016 and TTI-017. These new lyophilized products are stable at 25 °C for at least six months or longer, and show excellent stability at 2–8 °C. Furthermore, each new formulation shows better stability than simply lyophilizing caspofungin itself (Table 2). Of interest is the improved stability of TTI-016 and TTI-017 compared to TTI-014. All of these formulations have a 4:1 ratio of PVP:caspofungin. However, the lyophile diluent used is different; for TTI-016, normal saline, TTI-017, PBS and TTI-014, water. The solid-state stability of both TTI-016 and TTI-017 is superior to TTI-014, and this can be attributed to the presence of salts in the lyophilized cake matrix. These salts have a favorable effect on stability and appear to help retard the formation of caspofungin hydrolysis products arising from residual water present.

Most importantly the reconstituted solutions for TTI-016 and TTI-017 are stable at 25 °C for at least 7 h (Table 4) versus 1 h only for CANCIDAS^®^, which may be problematic for an IV drug administered over 1 h in the hospital. At this time, it is not clear why the PVP formulation has superior reconstituted stability, but perhaps PVP is closely associated with caspofungin in solution and interferes with hydrolysis. This is under ongoing investigation.

A further criterion that may improve the efficacy of inhaled antifungal treatment is to align the aerosol particle size with the fungal spore size. Both *Aspergillus* and *Pneumocystis* spores are about 2–3 microns [1,16]. These particle sizes allow the spores to reach the alveoli in the lower airway. The new formulations are suitable for aerosolization and have the proper particle size to achieve the deep lung penetration that is associated with activity at the infection site.

CANCIDAS^®^ clinical efficacy with the current IV dosing may not be high enough to achieve significant distribution to the lung. Increasing the IV dose is limited by liver toxicities due to systemic distribution. The low level of drug distribution to the lung by IV delivery may be overcome by aerosolized caspofungin administered directly to the lung. In the current study, the C_max_ and AUC with the inhaled TTI-016 is 31-fold and 47-fold higher than with IV delivery, respectively (Table 6 and Figure 1). Moreover, TTI-016 achieves 60–70% higher C_max_ and AUC in the lungs than the aerosolized API alone (Table 6). This may be due to improved aerosol characteristics and lung deposition with TTI-016. In addition to achieving superior lung AUC and C_max_ with the inhaled drug, significant differences in the drug’s half-life were also observed when compared to IV administration. The plasma half-life of IV caspofungin is about 11 h, but the half-life of inhaled drug, as measured in the lung, is 18 h for IV CANCIDAS^®^ versus 32 h with TTI-016. The difference in lung half-life between IV and inhaled TTI-016 is significant, and it is likely due to metabolizing enzymes found in plasma that are not present in the lung cavity. The aerosol delivery of TTI-016 deposits drug directly to the lung cavity. This is important as the site of action for inhibiting beta glucan synthase activity and hyphae growth and propagation is within the lung cavity. The long lung half-life may also provide an opportunity for smaller dosage and less frequent dosing schedule, which further benefit patients without sacrificing efficacy.

The targeted caspofungin MEC_90_ for *Aspergillus* was derived from a study by the Transplant-Associated Infection Surveillance Network, where 288 *Aspergillus* isolates showed that more than 95% of these were susceptible to a drug concentration of 0.25 µg/mL [17]. Our data indicated that a single dose of inhaled TTI-016 maintained drug concentrations above this MEC for 7 days, where an IV CANCIDAS^®^ dose could not (Figure 1a). The inhaled TTI-016 may be suitable for once-a-week administration. This weekly dosing schedule may not only provide superior coverage but also reduce patient burden in administrating caspofungin. Furthermore, the inhaled formulation and delivery could also prove useful for prophylaxis whereas daily IV delivery is not. The inhaled TTI-016 also reduced systemic drug exposure when compared to IV CANCIDAS^®^, as shown in Figure 1b–d. Inhaled TTI-016 minimized drug distribution to the liver and is remarkably similar in profile to the inhaled API (Figure 1d). The liver C_max_ for CANCIDAS^®^ IV administered daily is at 24 h after dosing (Figure 1d). Therefore, drug continuously accumulates in the liver during the treatment period and this can lead to safety concerns, particularly when IV doses used are higher than the FDA approved dose to maximize efficacy. A balance of drug concentrations in lung and liver must be reached to accomplish the best clinical efficacy and safety profile. A once-a-week inhaled stable formulation of caspofungin may deliver an efficacious dose of drug to the lung, while minimizing liver exposure with its subsequent toxicities.

**Figure 3:**
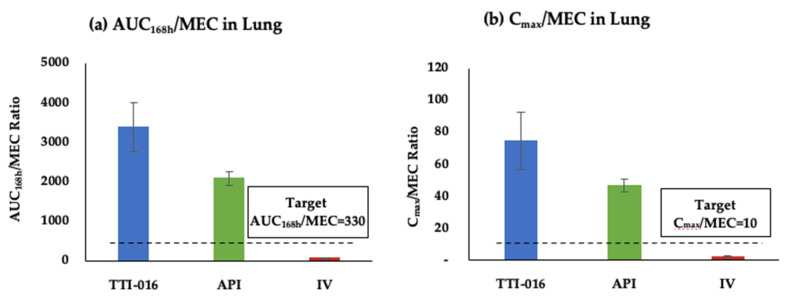
Comparison of PK/PD parameters in the lung between IV CANCIDAS^®^ and aerosolized TTI-016 and API. (**a**) AUC/MEC ratio in lung of inhaled TTI-016, inhaled API and IV CANCIDAS^®^. (**b**) C_max_/MEC ratio in lung of inhaled TTI-016, inhaled API and IV CANCIDAS^®^. The dashed line indicates the PK/PD index target level.

The clinical efficacy of caspofungin in treating invasive aspergillosis is highly predictable with defined PK/PD parameters, C_max_/MEC and AUC/MEC ratios [18,19]. Invasive aspergillosis rodent models show that the animal survival and lung fungal burden reduction is achieved when C_max_/MEC ratio exceeds 10 and AUC/MEC ratio is over 330 [19]. In our study, the IV cohort did not attain the targeted PK/PD indices and predicted a weak human clinical efficacy profile (Figure 3). Several clinical trials with the standard doses of IV CANCIDAS^®^ only showed approximately 40% human efficacy against invasive aspergillosis [7]. Based on the PK/PD indices, these patients were under dosed because of toxicity concerns at higher doses, and it is not surprising that better clinical efficacy is unattainable with IV administration. However, the inhaled TTI-016 cohort showed C_max_/MEC and AUC/MEC ratios in the lung at 73 and 3376, respectively, which are indices well-above the target levels to provide clinical benefit (Figure 3). These findings support further investigation, pre-clinical inhalation toxicology studies and clinical development.

## 5. Conclusions

Here, we described a new inhaled formulation of caspofungin that has good solid state and reconstituted stability at room temperature. Most importantly the new formulation also increased lung drug half-life and lung exposure without increasing systemic exposure. This is critical for antifungal drug development where toxicity is often a limiting factor for human efficacy. We have addressed this issue by formulation and delivery. By direct administration to the lung, the site of fungal infection, the drug distribution characteristics met the pharmacodynamic indices associated with clinical benefit. For both PCP caused by *Pneumocystis jiroveci* and IPA caused by *Aspergillus* that exist almost exclusively in the host’s lung alveoli and complete their life cycle stages in the lungs makes inhaled delivery an option for treating these diseases. The benefit to patients of the inhaled room temperature-stable formulations is the ability overcome the current challenges of antifungal drug therapy. The results illustrate how reformulation and targeted aerosol delivery may improve efficacy for the well-known antifungal drug caspofungin. Further animal toxicology studies may be pursued to support the clinical trials.

## 6. Patents

Some of this work described has been filed internationally, PCT/US2019/017520.

## Figures and Tables

**Table 1:** MIC or MEC of various formulations against selected fungal species.

			MIC or MEC, µg/mL				
No.	Species	Strain ID	CANCIDAS^®^	TTI-016	TTI-017	Caspofungin Diacetate	Amphotericin B
1	*Candida albicans*	ATCC44858	0.5	0.25	0.25	0.25	0.125
2	*Candida albicans*	ATCC90028	0.25	0.25	0.25	0.25	0.125
3	*Candida albicans*	ATCC90028 + 50%human serum	0.125	0.25	0.25	0.125	0.25
4	*Candida albicans*	Azole-R (20183.073)	0.25	0.25	0.25	0.25	0.125
5	*Candida albicans*	Azole-R (20186.025)	0.5	0.5	0.25	0.25	0.25
6	*Candida glabrata*	ATCC 36583	0.25	0.5	0.25	0.25	0.125
7	*Candida krusei*	ATCC 6258	1	1	1	1	0.25
8	*Candida parapsilosis*	ATCC 22019	1	2	1	1	0.25
9	*Candida tropicalis*	ATCC 200956	0.5	0.5	0.25	0.25	1
10	*Aspergillus fumigatus*	ATCC 13073	0.125	0.125	0.125	0.0625	0.25
11	*Aspergillus fumigatus*	ATCC204305	0.125	0.25	0.125	0.125	0.5
12	*Trichophyton rubrum*	ATCC 10218	0.25	0.25	0.25	0.125	0.125

MIC minimum inhibitory concentration; MEC minimum effective concentration.

**Table 2:** Caspofungin formulation stability at storage at 5 °C and 25 °C.

PVP:Caspofungin Diluent Time (Months)		5 °C				25 °C			
	API (Lyo Process)	TTI-013	TTI-014	TTI-016	TTI-017	TTI-013	TTI-014	TTI-016	TTI-017
	0:1	2:1	4:1	4:1	4:1	2:1	4:1	4:1	4:1
	Water	Water	Water	N-Saline	PBS	Water	Water	N-Saline	PBS
	HPLC Purity %								
0	95.42	98.97	99.14	99.73	99.72	98.97	99.14	99.73	99.72
0.5	--	--	--	99.64	99.62	--	--	99.40	99.48
1	--	--	--	99.55	99.61	--	--	99.23	99.36
2	--	98.46	99.03	99.59	99.58	96.63	97.73	98.88	98.94
3	--	--	--	99.50	99.63	--	--	98.06	98.32
6	--	--	--	99.54	99.52	--	--	97.99	98.21
12	--	--	--	99.14	99.39	--	--	97.14	96.45

Caspofungin amount of each formulation was measured by HPLC and presented as purity %.

**Table 3:** Comparison of total impurities of CANCIDAS formulation and TTI-016 and TTI-017 at 25 °C.

Time (Weeks)	Total Impurities %		
	CANCIDAS Formulation ^a^	TTI-016 **	TTI-017 **
0	1.93	0.27	0.28
2	2.06	0.60	0.52
4	2.09	0.77	0.64

^a^ US patent 9636407 B2 [13]. The double asterisk (**) corresponds to significant difference (*p* < 0.01) compared to CANCIDAS formulation.

**Table 4:** HPLC purity of reconstituted solution stability of TTI-016 and TTI-017 at 25 °C.

Time (h)	HPLC Purity %	
	TTI-016	TTI-017
0	99.34	99.38
1	99.25	99.33
4	99.00	99.04
7	98.93	98.73

**Table 5:** Aerosol properties of formulations.

Formulation Compositions					
Solution Number	API	TTI-002	TTI-013	TTI-016	N-Saline
Caspofungin diacetate final concentration, mg/mL	10	10	10	10	0.9% Saline
PVP concentration final concentration, mg/mL	0	10	20	40	
PVP K30 /Caspofungin diacetate ratio (*w/w*)	0:1	1:1	2:1	4:1	
Caspofungin diacetate Stock (100 mg/mL) in mL		1	1	1	
PVP K30 Stock (100 mg/mL) in mL		1	2	4	
0.9% normal saline (approx. volumes) in mL		8	7	5	
Total (fill to mark in 10 mL vol flask w saline)		10 mL	10 mL	10 mL	
**Aerosolization Data**					
Aerosolization time, min (A)	12	12	12	12	12
Pre wt, g (nebulizer + formulation), B		44.8	42.8	42.9	44.7
Post wt, g (nebulizer + formulation), C		43.0	41.0	41.3	42.6
Dispersion rate, mg/min = (B − C)/A (Calculation carried out with unrounded data for pre- and post-weights)		149.6	151.2	137.8	169.4
**Aerosol Particle Size Distribution**					
MMAD ^a^, microns	1.15	0.89	1.09	1.10	0.52
GSD ^b^	2.67	3.4–3.8	3.1–3.7	4.6–5.6	2.2–3.3

^a^ Mass Median Aerodynamic Diameter, ^b^ Geometric Standard Deviation.

**Table 6:** Caspofungin concentration in rat tissues following inhaled delivery with TTI-016 and API and intravenous delivery with CANCIDAS^®^.

Time (h)	Lung (µg/g)			Plasma (µg/mL)			Kidney (µg/g)			Liver (µg/g)		
	TTI-016	API	CANCIDAS	TTI-016	API	CANCIDAS	TTI-016	API	CANCIDAS	TTI-016	API	CANCIDAS
0.5	16.17 ± 4.41	10.83 ± 0.81	0.58 ± 0.07	2.2 ± 0.32	2.03 ± 0.35	11.03 ± 0.47	0.11 ± 0.05	0.16 ± 0.02	0.50 ± 0.00	0.04 ± 0.00	0.09 ± 0.01	0.25 ± 0.04
2	18.43 ± 4.37	10.37 ± 2.15	0.54 ± 0.04	2.25 ± 0.46	1.93 ± 0.25	7.72 ± 0.51	0.16 ± 0.03	0.16 ± 0.02	0.67 ± 0.13	0.09 ± 0.02	0.13 ± 0.01	0.31 ± 0.02
24	8.76 ± 1.54	5.31 ± 0.40	0.18 ± 0.01	0.32 ± 0.16	0.23 ± 0.10	0.80 ± 0.06	0.16 ± 0.07	0.16 ± 0.07	0.50 ± 0.05	0.29 ± 0.15	0.3 ± 0.06	0.66 ± 0.02
48	5.4 ± 1.37	3.37 ± 0.95	0.09 ± 0.01	0.09 ± 0.01	0.08 ± 0.01	0.19 ± 0.03	0.07 ± 0.00	0.12 ± 0.01	0.33 ± 0.02	0.13 ± 0.01	0.18 ± 0.04	0.45 ± 0.07
168	0.36 ± 0.11	0.39 ± 0.05	0.00 ± 0.00	0.01 ± 0.00	0 ± 0.00	0.00 ± 0.00	0.01 ± 0.00	0.00 ± 0.03	0.07 ± 0.01	0.03 ± 0.01	0.02 ± 0.03	0.11 ± 0.01

**Table 7:** Caspofungin pharmacokinetics in IV CANCIDAS^®^, inhaled API and inhaled TTI-016. The data are presented as the mean values (*n* = 3) ± SD.

	Rat Pharmacokinetic Parameters					
	Plasma			Lung		
	IV CANCIDAS^®^	Inhaled API	Inhaled TTI-016	IV CANCIDAS^®^	Inhaled API	Inhaled TTI-016
AUC (24 h) (µg·h·mL^−1^)	85.93 ± 2.96	21.14 ± 1.31 **	25.43 ± 3.60 **	8.88 ± 0.72	191.02 ± 27.97 *	329.07 ± 68.74 *
AUC (48 h) (µg·h·mL^−1^)	97.72 ± 3.32	24.84 ± 2.54 **	30.44 ± 5.43 **	12.12 ± 0.88	295.14 ± 22.02 *	498.97 ± 93.09 *
AUC (168 h) (µg·h·mL^−1^)	108.96 ± 1.60	29.45 ± 3.17 **	36.84 ± 5.32 **	17.54 ± 1.06	520.98 ± 43.09 *	844.05 ± 154.79 *
Kel (h^−1^)	0.07 ± 0.01	0.05 ± 0.01	0.06 ± 0.01	0.04 ± 0.00	0.02 ± 0.00	0.02 ± 0.00
t 1/2 (h)	10.29 ± 0.84	13.3 ± 2.54	11.78 ± 1.71	18.00 ± 1.00	38.67 ± 2.52	31.67 ± 3.51
C_max_ (µg/mL)	11.03 ± 0.47	2.04 ± 0.33	2.26 ± 0.36	0.58 ± 0.07	10.83 ± 0.81	18.43 ± 4.37
T_max_ (h)	0.5	1	1	0.5	0.5	2

The asterisk (*) corresponds to significant difference (*p* < 0.05); the double asterisk (**) represents significant difference (*p* < 0.01) compared to IV CANCIDAS^®^.

## Data Availability

The data presented in this study are available in Supplementary Material.

## Supplementary Materials

The following are available online at https://www.mdpi.com/article/10.3390/pharmaceutics13040504/s1, Table S1: HPLC Gradient; Table S2: Compositions of Pre-lyophilized Formulations; Table S3: HPLC Gradient; Table S4: Caspofungin acetate lyo solution; Table S5: pH of PVP K30 Stock Solutions in DI Water, 0.9% Saline and PBS Buffer; Table S6: Lyophilized Caspofungin Diacetate Formulations Prepared from Water, Saline and PBS; Table S7: Target Dose and Delivery Route for the Exposure Groups; Table S8: HPLC Condition.
