# Supplementary material for: Assessment and Development of the Antifungal Agent Caspofungin for Aerosolized Pulmonary Delivery
Source: Pharmaceutics. 2021 Apr 7;13(4):504. doi: 10.3390/pharmaceutics13040504 (PMC8067632; doi:10.3390/pharmaceutics13040504)
Supplement: Supplementary file 1 [file pharmaceutics-13-00504-s001.pdf]

pharmaceutics

Supplementary Materials: Assessment and Development of the Antifungal Agent Caspofungin for Aerosolized Pulmonary Delivery

Iching G. Yu and David M. Ryckman

S1. In Vitro Testing, Test Article Preparations

1.1. CANCIDAS®

The 5 mg/mL stock solution was prepared as the following. The refrigerated vial was equilibrated to room temperature. Test vial was reconstituted aseptically with 10.5 mL 0.9% NaCl. The powder was dissolved completely, mixing until a clear solution was obtained. The final concentration was 5.0 mg/mL of caspofungin freebase. The reconstituted solution was stable for 1 hr at room temperature. The 5 mg/mL stock solution was then diluted in 0.9% NaCl to prepare the 3.2 mg/mL working stock solution for MIC testing.

1.2. Formulations TTI-016 and TTI-017

The refrigerated vial was equilibrated to room temperature. The powder was dissolved completely, mixing until a clear solution was obtained. Test vial was reconstituted aseptically with 1.8 mL 0.9% NaCl. The white to off-white powder was dissolved completely, mixing gently until a clear solution was obtained. The final concentration was 5.0 mg/mL of caspofungin freebase. The reconstituted solution was used within 1 hr at room temperature. The 5 mg/mL stock solution was then diluted with 0.9% NaCl to prepare the 3.2 mg/mL working stock solution for MIC testing.

1.3. Caspofungin Diacetate

The 5 mg/mL API stock solution was prepared as the following. The refrigerated vial was equilibrated to room temperature. Test vial was filled aseptically with 1.8 mL 0.9% NaCl. The powder was dissolved completely, mixing until a clear solution was obtained. The final concentration was 5.0 mg/mL of caspofungin freebase. The reconstituted solution was used within 1 hr at room temperature. The 5 mg/mL stock solution was then diluted with 0.9% NaCl to prepare the 3.2 mg/mL working stock solution for MIC testing.

1.4. Amphotericin B

g compound, corresponding to 1.2 mg active compound with a correction factor of 2.40 was added 0.375 mL of water for injection to generate the 3.2 mg/mL working stock solution for testing. The 3.2 mg/mL working stock solution was 2-fold diluted serially in 0.9% NaCl to prepare a total of eleven 50-fold stock solutions, concentrations ranging from 3.2 to 0.003 mg/mL. A 4 µL aliquot of each dilution was added to 196 µL of broth medium seeded with the organism suspension in wells of a 96 well plate (fungal cell count: 1 x 10^3 to 1 x 10^4 colony forming units per mL). The final volume was 200 µL in each well.

S2. Lyo Process, Stability and Analysis

S2.1. HPLC Analysis

HPLC analysis was carried out on a Waters 2695 separations module equipped with an autosampler and a Waters 996 photodiode array detector. The HPLC method is described below:

Column: Waters symmetry C18 3.5 µm, 4.6 x 75 mm

Flow rate: 1.0 mL/min

Detection: 220 nm

Column Temperature: 30 °C

Autosampler temperature: 4 °C

Injection volume: 50 µL

Run time: 70 min

Mobile phase: A: Add 1.0 mL of perchloric acid and 0.75 g of sodium chloride in 1000 mL HPLC grade water; B: Acetonitrile.

Gradient: As shown in Table S1

Table S1. HPLC Gradient.

Time (min)	Mobile Phase A (%)	Mobile Phase B (%)
0	67	33
14.5	67	33
35	50	50
45	25	75
50	20	80
52	20	80
53	67	33
70	67	33

S2.2. Preparation of Lyophilized Caspofungin Diacetate Formulations with PVP/API Ratio of 2:1 and 4:1

The liquid formulations to be lyophilized were prepared according to Table S2. Aliquots of 0.5 mL of each liquid formulation were placed in 3 mL glass vials, frozen on dry ice for 40 min. The vials were partially stoppered with lyo septa and lyophilized for 5 days. The vials were placed on stability at 5 °C and 25 °C (dark).

Table S2. Compositions of Pre-lyophilized Formulations.

	API	TTI-013	TTI-014
Caspofungin diacetate 160 mg/mL stock	0.25 mL	0.625 mL	0.625 ml
PVP K30 100 mg/mL stock pH 6.05 adjusted with 1 N NaOH	--	2.0 mL	4.0 mL
Deionized water	1.75 mL	2.375 mL	0.375 mL
Total volume	2.0 mL	5.0 mL	5.0 mL
PVP K30 /Caspofungin diacetate ratio (*w/w*)	0	2:1	4:1

S2.3. Caspofungin Acetate Lyo Formulation Preparation and Assay by HPLC

S2.3.1. Preparation of 20 mg/mL Caspofungin Acetate Pre-Lyo Solution (Caspofungin Acetate/PVP=1:4)

Caspofungin acetate (100 mg, 97.6% assay) was dissolved in DI water (0.525 g) to obtain 0.625 g caspofungin acetate stock solution at a nominal concentration of 160 mg/mL (assuming density = 1). PVP K30 (400 mg) was dissolved in PBS buffer (3.6 g), adjust pH to 6 with 25 µL of 1.0 N HCl to obtain 4.0 g of PVP stock solution with a nominal

concentration of 100 mg/mL (assuming density = 1). Caspofungin acetate stock solution of step 1 was combined with the PVP solution of step 2. An additional 0.375 g of PBS buffer was added to obtain 5.0 g of the pre-lyo solution with a nominal caspofungin acetate concentration of 20 mg/mL (assuming density = 1). The theoretical wt% caspofungin acetate concentration is 100 × (100 × 97.6%) / 5000 = 1.952%

S2.3.2. Preparation of Caspofungin Acetate Lyo Formulation Based on Weight of the Pre-Lyo Solution (0.5 g/vial)

0.5 g of the pre-lyo solution was weighed into a lyo vial. The vial was frozen on dry ice for 1 hr and lyophilized for 5 days as described above.

S2.3.3. Preparation of Caspofungin Acetate Lyo Formulation Based on Volume of the Pre-Lyo Solution (0.5 mL/vial)

0.5 mL of the pre-lyo solution was pipetted into a lyo vial. The vial was frozen on dry ice for 1 hr and lyophilized for 5 days as described above.

S2.3.4. HPLC Assay

HPLC analysis was carried out on a Waters 2695 separations module equipped with an autosampler and a Waters 996 photodiode array detector. The HPLC method is described below:

Column: Waters Symmetry C18 Column, 3.5 μm, 4.6 mm × 100 mm
UV detection: 220 nm
Column temperature: 30 °C
Flow rate: 1.0 mL
Injection volume: 50 μL
Sample tray temperature: 4 °C
Run time: 70 min
Mobile phase:
Mobile phase A: Add 1.0 mL of perchloric acid and 0.75 g of sodium chloride, q.s. with water to 1000 mL.
Mobile phase B: Acetonitrile
Gradient program: As shown in Table S3.

Table S3. HPLC Gradient.

Time (min)	Mobile Phase A (%)	Mobile Phase B (%)
0	67	33
14.5	67	33
35	50	50
45	25	75
50	20	80
52	20	80
53	67	33
70	67	33

S2.3.5. Preparation of Caspofungin Acetate Standard Solution

Corrected assay: (100 − 2.7) × 99.3% = 96.6%. In a 25.00 mL volumetric flask was placed 13.78 mg of the caspofungin acetate working standard. DI water was added to dissolve and q.s. to mark.

S2.3.6. Preparation of HPLC Sample of Pre-Lyo Solution

23.17 mg of the pre-lyo solution was weighed into an HPLC vial. DI water was added to give a total weight of 1.000 g (1.000 mL) of solution.

S2.3.7. Assay Calculation for Pre-Lyo Solution

Calculation of Caspofungin acetate concentration in HPLC sample:

Caspo conc. = (Area $_{sample}$/Area $_{std}$)xcorr. Assay $_{std}$xW_{std}/V_{std} = (14974300 / 18005050) × 96.6% × 13.78 / 25 = 0.44 mg/mL

Caspofungin acetate concentration (wt%) in pre-lyo solution:

Caspofungin acetate concentration (wt%) = (Caspofungin acetate conc. of HPLC sample x volume of HPLC sample)/Weight of Pre-lyo solution) × 100 = (0.44 × 1.0 / 23.17) × 100 = 1.90%

% Lyo solution concentration = 100x(1.90% / 1.978%) = 96.1%

S2.3.8. Reconstituted Caspofungin Acetate Lyo Vial for HPLC Analysis

Caspofungin acetate lyo cake (50 mg) was reconstituted by adding 1.0 mL of DI water via a pipette to obtain stock solution. The solution was vortexed for 20–30 seconds and visually checked to ensure complete dissolution. 55 µL of the stock solution was pipetted and added into a HPLC vial containing 1.0 ml of DI water. The solution was vortexed for a few seconds to mix.

S2.3.9. Preparation of Caspofungin Acetate Standard Solution

Corrected assay: (100 − 2.7) × 99.3% = 96.6%. In a 25.00 mL volumetric flask was placed 13.95 mg of the caspofungin acetate standard. DI water was added to dissolve and q.s. to mark.

S2.3.10. Assay Calculation for Lyo Cake

Lyo cake based on 0.5 g of the pre-lyo solution:

Theoretical amount of caspofungin acetate (mg)/vial = 0.5 × 1000 × 1.90% = 9.5 mg

Step 1: Calculation of Caspofungin acetate concentration in HPLC sample

HPLC sample Caspofungin acetate conc. = (Area $_{sample}$/Area $_{std}$)xcorr. Assay $_{std}$xW_{std}/V_{std} = (15655992 / 17947658) × 96.6% × 13.95 / 25 = 0.47 mg/mL

Calculation of caspofungin acetate in lyo vial:

Weight of caspofungin acetate in lyo vial = (HPLC sample Caspofungin acetate conc. of step 1 x Volume of HPLC sample/Volume of reconstituted solution used to make HPLC sample x total volume of reconstituted solution = (HPLC sample Caspofungin acetate conc. of step 1 × 1.055 / 0.055) × 1.050 = (0.47 × 1.055 / 0.055) × 1.050 = 9.47 mg

Calculation of caspofungin acetate assay:

Assay =100x(weight by HPLC/label claim)= 100x(9.47 / 9.5) = 99.7%

Table S4 shows details of a caspofungin acetate lyo solution prepared as described herein.

Table S4. Caspofungin acetate lyo solution.

	Amount of Lyo Solution	Theoretical	Results Based on HPLC Assay	%Theoretical
Caspofungin acetate Lyo solution 20 mg/mL	N/A	19.56 mg/mL (adjusted for assay)	19.0 mg/mL	97.1

S2.4. Preparation of Lyophilized Caspofungin Diacetate Formulations (Caspofungin Diacetate/PVP K30 = 1:4) in Seionized Water, 0.9% Saline and PBS Buffer

S2.4.1. Preparation of caspofungin diacetate stock solution

Caspofungin diacetate (0.8 g) was dissolved in deionized water (4.2 g) to obtain 5.0 g of a clear solution with caspofungin diacetate concentration of 160 mg/ml (assuming density = 1 mg/mL).

S2.4.2. Preparation of PVP K30 Stock Solution in Seionized Water, 0.9% Saline and PBS Buffer

PVP K30 (0.8 g) was dissolved in a diluent (7.2 g) as shown in Table S5 to obtain a clear solution. The pH of the resultant solution was adjusted to about 6 with 1N NaOH solution.

Table S5. pH of PVP K30 Stock Solutions in DI water, 0.9% Saline and PBS buffer.

Diluent	pH
Deionized water	6.03
0.9% saline	6.20
PBS buffer	5.95

S2.4.3. Preparation of Liquid Formulations for Lyophilization

Caspofungin diacetate/PVP K30 formulations: The caspofungin diacetate stock solution of Part A (1.25 mL), the PVP K30 stock solution (8.0 g) and the corresponding diluent in the PVP K30 solution (0.75 mL) of Part B was combined to obtain 10 mL of the liquid formulations ready for lyophilization. The ratio of caspofungin diacetate/PVP K30 is 1:4 (*w/w*).

Caspofungin diacetate only (control): Caspofungin diacetate (0.2 g) was dissolved in deionized water (9.8 mL) to obtain a clear solution with caspofungin diacetate concentration of 20 mg/mL.

Compositions of each formulation were shown as Table S6.

Table S6. Lyophilized Caspofungin Diacetate Formulations Prepared from Water, Saline and PBS.

	TTI-015	TTI-016	TTI-017
Caspofungin diacetate (mg)	10	10	10
PVP K30 (mg)	40	40	40
Total Volume (mL)	0.5	0.5	0.5
Acetate buffer pH	6.13	6.01	6.14
PVP K30 /Caspofungin diacetate ratio (*w/w*)	4:1	4:1	4:1

S2.4.4. Lyophilization

Fourteen 0.5 mL aliquots of each liquid formulation prepared in part C were placed in 14 3-mL glass vials. The vials were frozen on dry ice for about 1 h and lyophilized for 5 days to obtain white cake. The vials were stoppered, crimp sealed and placed on stability at −20 °C, 5 °C, and ambient (dark) conditions. Lyophilization was accomplished using a VirTis benchtop manifold lyophilizer under vacuum of <50 mTorr.

S2.4.5. HPLC Analysis

One vial of the lyophilized cake was reconstituted in 1 mL of deionized water to obtain a 10 mg/mL caspofungin diacetate solution. An aliquot (55 μL) was removed and diluted with 1 mL of deionized water for HPLC analysis.

S3. Aerosol Characterization

The stock solutions of caspofungin diacetate (100 mg/mL) and polyvinylpyrrolidone (PVP K30, 100 mg/mL) were prepared. In addition, 0.9% saline was used to generate aerosols for the purpose of comparing particle size.

S3.1. Preparation of Caspofungin Diacetate Stock Solution:

500 mg of caspofungin diacetate was dissolved in 5.0 mL of 0.9% saline to obtain 5 mL of caspofungin diacetate stock solution. The concentration is 100 mg/mL (assuming the density of the stock solution was close to 1).

S3.2. Preparation of PVP K30 Stock Solutions:

In a 25 mL volumetric flask 2.5 g of PVP K30 was dissolved in 20 mL 0.9% saline. Adjusted the pH to 6 with 1N NaOH solution. Normal saline was added to the mark and mixed well. This provided 100 mg/mL PVP K30 stock solution.

Temperature, Relative Humidity and Airflow Rate: Inhalation exposure chamber temperature, relative humidity and airflow rate (liters per minute; LPM) were measured and recorded once during the exposure. The chamber temperature and relative humidity were monitored with a hand-held thermohygrometer (35612 series, Oakton Instruments, Vernon Hills, IL).

S4. Inhalation Pharmacokinetics

Caspofungin was administered to rats at a target dose of 2 mg/kg by nose only inhalation (bydeposition) or intravenously (IV) to determine the plasma and tissue concentrations and pharmacokinetics.

S4.1. Animal Preparation

Sprague-Dawley derived rats [Crl:CD®(CD)Br] were obtained from Charles River Laboratories, Inc., Wilmington, MA, for use in this study. The rats were 52 days of age (approximately 7.5 weeks) upon arrival. The animals were held in quarantine for 7 days prior to administration of the test article. The test animals were approximately 8.5 weeks old at the start of the first exposure to the test article. To condition the animals to placement and restraint in the nose-only exposure system and reduce stress during the exposure phase, the animals were acclimated to the holding tubes by placing each rat in a nose-only holding tube for approximately 45 minutes one working day prior to exposure.

S4.2. Test Article Preparation for API

For inhalation exposure, a 10 mg/mL dosing solution was prepared by dissolving 800 mg of caspofungin diacetate powder in 80 mL of 0.9% saline solution. The resulting solution was aseptically filtered and kept refrigerated between 2–8 °C until used. The formulation was aerosolized for inhalation administration.

S4.3. Test Article Preparation for TTI-016

S4.3.1. Preparation of Caspofungin diacetate stock solution

800 mg of caspofungin diacetate, which was warmed from storage as previously described, was dissolved in in 8.0 mL of 0.9% saline to obtain 8 mL of caspofungin diacetate stock solution. The concentration should have been close to 100 mg/mL (assuming the density of the stock solution was close to 1).

S4.3.2. Preparation of PVP K30 Stock Solution

In a 50 mL volumetric flask, 5.0 g of PVP K30 was dissolved in 45 mL of 0.9% saline. The pH was adjusted to 6 with 1N NaOH solution dropwise. Normal saline was added to the mark, approx 5 mL, and was mixed well to give a total volume of 50 mL. This provided 100 mg/mL PVP K30 stock solution.

S4.3.3. Preparation of Test Article: Caspofungin Diacetate (10 mg/mL), PVP K30 (40 mg/mL)

In a 100 mL flask or glass bottle, 8.0 mL of caspofungin diacetate stock solution and 32 mL of PVP stock solution were added. 40 mL of normal saline was added in the flask and gently well mixed. The solution was sterile filtered through a 0.2-micron filter using a slight vacuum into a sterile 100 mL flask or bottle. The flask was stoppered. The test article was stored at 4 °C until use.

S4.4. CANCIDAS® Solution for IV Administration (IV)

A 2 mg/mL dosing solution from commercially obtained CANCIDAS® (containing 54.6mg of caspofungin diacetate) was prepared by adding 10.8 mL of 0.9% saline into the CANCIDAS® vial and swirling gently until the powder dissolved. 10.0 mL of this solution was extracted and added a 25 mL volumetric flask which was diluted to the mark with 0.9% saline and mixed well. The resulting solution was aseptically filtered and kept refrigerated between 2–8 °C until used.

S4.5. Test Article Dosing

The animals were randomized into 15 animals per group based on body weight. Each group was dosed as shown in Table S7 below.

Table S7. Target Dose and Delivery Route of the Exposure Groups.

Exposure Group	Target Dose	Route	Duration (minutes)	Number of Animals
I	2 mg/kg	Inhalation (API)	125	15
II	2 mg/kg	Inhalation (TTI-016)	140	15
III	2 mg/kg	IV (CANCIDAS®)	-	15

S4.6. Inhalation Exposure Methods

The inhalation exposure part of the study was conducted in an inhalation facility. The supply air to the laboratories was preconditioned and automatically controlled with a thermostat and humidistat. Each flow-past nose-only inhalation exposure chamber (Lab Products Inc., Seaford, DE) is comprised of 52 ports. The chambers were encased in an acrylic enclosure to isolate the exposure chamber and protect laboratory personnel. The test atmosphere inlet and exhaust configurations provided a uniform and continuous stream of fresh test atmosphere to the animals undergoing exposure. After flowing out of the supply port, any excess test atmosphere, along with exhaled air, is drawn into the chamber exhaust manifold without entering other ports.

During the inhalation exposure, the animals were restrained in nose-only holding tubes (CH Technologies, USA, Westwood, NJ). Following confirmation of the correct animal number, each tube was placed in a pre-designated port of the inhalation exposure chamber. Chamber ports were rotated for each exposure; placement for each exposure is documented in the study records. Animal tube loading and unloading and tube insertion and removal from the chamber manifold processes were performed according to laboratory standard operating procedures that are designed to minimize stress to the rats. The

rats were observed frequently while restrained to ensure that they remained in the tubes and were not in danger of injury. At the end of each exposure, when the chamber was purged of the test substance (less than one minute), the tubes with the animals were removed. The rats were removed from the tubes and returned to their home cages. The holding tubes were sanitized after each use.

S4.6.1. Test Atmosphere Generation

Test atmosphere at the desired concentrations was generated by aerosolizing the test substance and mixing it with compressed filtered air to produce a continuous supply of test atmosphere. Test atmospheres were generated by aerosolizing the test formulation with a commercially available nebulizer using compressed air of breathable quality and which is filtered with a compressed air filter and a carbon adsorber. Exhaust from the exposure chambers was moved through a high efficiency particulate air (HEPA) filter by a ring compressor and exhausted outside the building. Inlet and exhaust flows to and from the chamber were controlled and continuously monitored by rotometers.

S4.6.2. Test Atmosphere Monitoring

Gravimetric Analysis: The test atmosphere concentration in the exposure chamber was determined gravimetrically each exposure by collecting test atmosphere samples on filters placed in closed-face filter holders in the breathing zone of the animals. The gravimetric sampling train consisted of a pre-weighed filter in series with a dry-gas meter connected to a constant flow vacuum pump. Samples were collected at a constant flow rate equal to the port flow of the delivery tube. The filter samples were weighed to determine the aerosol mass collected. The dry-gas meter measured the corresponding volume of chamber air sampled and the weight-to-volume ratio was determined to obtain the aerosol mass concentration.

S4.7. Intravenous Administration

Animals in the IV dosing group received a single injection via the tail vein at a dosing volume of 1 mL/kg.

S4.8. Toxicology Methods

Moribundity/Mortality Observations and Physical Examinations/Clinical Observations: Prior to initiation of dosing (exposure), animals were observed at least once daily for mortality or evidence of moribundity. A detailed, hand-held physical examination was conducted on all animals once during the quarantine period (prior to randomization). During the treatment period, the animals were observed daily for mortality or evidence of moribundity; these checks were separated by a minimum of four hours. Daily cage-side clinical observations were conducted during exposure, and daily hand-held clinical observations were conducted before and after exposure. Observations included, but were not limited to the following: changes in the skin and fur, eyes, and mucous membranes; effects on the respiratory, circulatory, autonomic and central nervous systems; and effects on somatomotor activity and behavior pattern.

Body Weights and Body Weight Changes: Body weights were determined one day after animal receipt; at randomization; and prior to exposure on Study Day 1, 2, 3 and 7 (as applicable based on scheduled euthanization).

Plasma and Tissue Samples/Necropsy: Whole blood samples were collected from three animals per time-point at approximately 0.5, 1, 2, 4, 8, 12, 24 and 48 hours and 7 days after dose administration for plasma drug level determination. Rats were anesthetized with 70% CO2/30% air and blood was collected from the retro-orbital plexus and placed into tubes containing anticoagulant (EDTA). Blood samples were placed on ice immediately following collection and processed (i.e., centrifuged) to plasma. The samples were

then stored frozen (at approximately −70 °C) until analyzed. All study animals surviving to scheduled necropsy were euthanized by an overdose of an intraperitoneal injection of sodium pentobarbital at 35–45 mg/kg. Tissue specimens (lung, liver and kidney) were collected from three animals per time point at 0.5, 2, 24 and 48 hours and 7 days after dose administration. All tissue specimens were stored frozen at approximately −70 °C until analyzed.

S4.9. Bioanalytical Method and Analysis

Calibration and Internal Standards: The reference standard, caspofungin acetate (lot number 02220902; Chunghwa Chemical Synthesis & Biotech, Taiwan), was stored at approximately −70 °C; and used without further purification for the preparation of calibration standards and quality control (QC) samples for the determination of caspofungin in plasma and tissue samples collected from this study. The internal standard (caspofungin acetate-d4; lot number 10-GJF-162-1) was stored at −20 °C.

Sample Preparation: For the determination of caspofungin in plasma, a 100 µL aliquot from each sample (in a 2 mL centrifuge tube) was mixed with 0.3 mL of acetonitrile (ACN; Spectrum, New Brunswick, NJ) containing 150 ng of internal standard. After shaking for five minutes, the sample was centrifuged at 4 ºC for 10 minutes to remove precipitated proteins and supernatant was transferred to an autosampler tube, diluted with 0.5 mL of water, and vortex-mixed for instrumental analysis.

For the determination of caspofungin in tissue, samples (lung – entire organ; liver – 1 gram; kidneys – one organ) were finely cut and extracted for analysis by adding 2.5 mL of ASTM Type I water and shaking for approximately 0.5 hour, after which 2.5 mL of acetonitrile (ACN; Spectrum, New Brunswick, NJ) were added following by shaking for another 0.5 hour. Subsequently, 100 µL of the supernatant was transferred to a 2 mL centrifuge tube and processed for analysis using the same procedure as for plasma.

Freshly prepared caspofungin standard curves and quality control (QC) samples were analyzed along with the study samples. Instrument calibrators and QC samples were prepared by adding 10 µL of a stock caspofungin solution in ACN/water (*v*/*v* 50/50) to 100 µL of blank rat plasma (for both plasma and tissue samples). Calibrator concentrations for plasma specimen analysis were approximately 0.050, 0.10, 0.20, 0.50, 1.0, 2.5, 5.0 and 10 µg/mL; QC samples were prepared at approximately 0.12, 4.0 and 8.0 µg/mL. Calibrator concentrations for tissue specimen analysis were approximately 1, 2, 5, 10, 20, 50 and 100 ng/sample; QC samples were prepared at approximately 2.4, 40 and 80 ng/sample. Calibrators and QC samples were processed for analysis following the procedure described above.

Analytical Equipment and Conditions: Calibrator, QC and study samples were analyzed under LC-MS-MS instrument conditions as detailed in Table S8.

Table S8. HPLC Condition.

**Instrument Operating Conditions SYSTEM: 4000 QTrap LC-MS-MS (AB SCIEX, Foster City, CA) Equipped with a 1200 HPLC (Agilent Technologies, Wilmington, DE)**			
HPLC CONDITIONS			
HPLC Column:		Kinetex Biphenyl 50 mm × 2.1 mm, 5µm, 100Å (Phenomenex, Torrance, CA)	
Column Temperature		25 °C	
Injection Volume:		5 µL	
Flow Rate:		300 µL/min	
Mobile Phase A:		0.1% formic acid in water	
Mobile Phase B:		0.1% formic acid in acetonitrile	
Program:	Time (minutes)	Mobile Phase A (%)	Mobile Phase B (%)
	0.00	70	30
	0.5	70	30
	1.0	5	95
	4.0	5	95
	4.1	70	30
	8.0	70	30
Run Time:		8 minutes	
Retention Time:		Caspofungin and Internal Standard – approximately 2.3 minutes	
MS-MS CONDITIONS			
Scan Type:		MRM	
Ion Source:		Turbo Spray ESI	
Ion Spray Voltage:		5500 Volts	
Polarity:		Positive	
Ion Source Temperature:		550 °C	
Collision Energy:		Caspofungin and Internal Standard: 20 Volts	
Ions monitored (Q1→Q3):		Caspofungin: 547.4 → 538.5; Internal Standard: 550.3→ 540.8	
Resolution:		Unit	
Data System:		Analyst® 1.6.3 (Applied Biosystems/MDS Sciex, Foster City, CA)	

The retention time of caspofungin was approximately 2.3 minutes. Calibration curves were calculated from the linear regression (weighting factor of $1/x^2$) of the caspofungin peak area to internal standard peak area ratios versus caspofungin concentration. Concentration of caspofungin in the samples was determined using the peak area ratio and the regression parameters of the calibration curve. Tissue results in ng were converted to µg/g using the amount of tissue extracted and the final extract volume.
